# Temporal variations in bacterial community diversity and composition throughout intensive care unit renovations

**DOI:** 10.1186/s40168-020-00852-7

**Published:** 2020-06-08

**Authors:** Jessica Chopyk, Kevan Akrami, Tovia Bavly, Ji H. Shin, Leila K. Schwanemann, Melissa Ly, Richa Kalia, Ying Xu, Scott T. Kelley, Atul Malhotra, Francesca J. Torriani, Daniel A. Sweeney, David T. Pride

**Affiliations:** 1 Department of Pathology, University of California, San Diego, USA; 2 Department of Medicine, University of California, San Diego, USA; 3 Department of Biology, San Diego State University, San Diego, USA

**Keywords:** Intensive care unit, Human microbiome, Microbial diversity, Built environment

## Abstract

**Background:**

Inanimate surfaces within a hospital serve as a reservoir of microbial life that may colonize patients and ultimately result in healthcare associated infections (HAIs). Critically ill patients in intensive care units (ICUs) are particularly vulnerable to HAIs. Little is known about how the microbiome of the ICU is established or what factors influence its evolution over time. A unique opportunity to bridge the knowledge gap into how the ICU microbiome evolves emerged in our health system, where we were able to characterize microbial communities in an established hospital ICU prior to closing for renovations, during renovations, and then after re-opening.

**Results:**

We collected swab specimens from ICU bedrails, computer keyboards, and sinks longitudinally at each renovation stage, and analyzed the bacterial compositions on these surfaces by 16S rRNA gene sequencing. Specimens collected before ICU closure had the greatest alpha diversity, while specimens collected after the ICU had been closed for over 300 days had the least. We sampled the ICU during the 45 days after re-opening; however, within that time frame, the alpha diversity never reached pre-closure levels. There were clear and significant differences in microbiota compositions at each renovation stage, which was driven by environmental bacteria after closure and human-associated bacteria after re-opening and before closure.

**Conclusions:**

Overall, we identified significant differences in microbiota diversity and community composition at each renovation stage. These data help to decipher the evolution of the microbiome in the most critical part of the hospital and demonstrate the significant impacts that microbiota from patients and staff have on the evolution of ICU surfaces.

Video Abstract

## Background

The most tenuous patients in a hospital are located in the intensive care unit (ICU). Critically ill patients are especially vulnerable to healthcare associated infections (HAIs), which represent a leading cause of death in the ICU [1]. Nearly 2 million hospitalized patients in the USA develop HAIs annually with an attributable mortality of 98,000 per year [2]. Multiple studies have sought to determine the role of hospital surfaces as a reservoir for healthcare-associated pathogens, though traditional culture techniques have failed to confirm the relationship between HAIs and the hospital environment [3, 4].

The development of 16S rRNA sequencing has made it possible to more completely characterize the breadth and diversity of different hospital microbial environments [5, 6]. Studies using 16S rRNA gene sequencing have shown that hospital environments are more diverse and dynamic than previously recognized and are affected by close contact with the human microbiome [7, 8]. An exhaustive study prospectively determined the microbiome of the wards in a newly completed hospital, finding a significant increase in human skin microbiota after hospital opening [9]. Other work has identified the durability of specific pathogens on hospital surfaces despite disinfection [10], which demonstrated that surface microbiomes vary depending on extent and diversity of human contact [11] and revealed the homogeneity of core microbiota across healthcare units [12]. These studies largely focused on hospital wards outside of the ICUs. Studies that have focused on ICUs have found increased abundances of skin-associated taxa [13], reduced diversity compared to non-patient care areas [8, 11, 14], and increased within species diversity compared to traditional culture techniques [8]. However, these studies have been somewhat limited in the number of specimens analyzed and duration of collection.

One of the most comprehensive culture independent studies to date was performed in a neonatal ICU (NICU) and focused on decontamination efforts to reduce HAIs. These efforts appeared to reduce certain taxa selectively while others that resembled gut microbiota remained intact [15–17]. While these findings highlight the interaction between the environment and neonates in the ICU, the taxa identified and microorganisms associated with HAIs are distinct from the adult ICU population [18, 19]. Thus, it is unclear how findings in the NICU relate to the adult ICU experience.

A critical step to understanding the relationship between the ICU environment and HAIs is the identification and analysis of activities that impact the ICU microbiome and its evolution. A prior study, using samples collected primarily from hospital wards, comprehensively characterized the microbiome of a newly opened hospital [9]. Renovation is a relatively common event as aging hospitals (and ICUs) are adapted to new patient needs and new technologies. The impact of renovation on the ICU microbiome has not been previously investigated to our knowledge. In this study, we prospectively examined the microbiota of surfaces at the interface of patients and healthcare workers. We obtained specimens from an ICU a week prior to renovation when patients and staff occupied the ICU, during renovation when there were no patients and staff present, and after renovation when patients and staff returned. Our goals were to characterize the evolution of the ICU microbiome in each of these various stages, identify factors that contribute to changes in the ICU microbiome, and evaluate sources that contribute to diversity and composition of the microbiome.

## Methods

### Sample collection

Samples were collected in an adult ICU in the Thornton Hospital in La Jolla, CA, between 11/16/2016 and 11/25/2017. Briefly, pre-moistened dual sterile swabs (BD Falcon Swube Screw Cap/Cotton Tip Applicators #281130) were wiped for 30 s over the surface of bedrails, keyboards, and sinks selected from six single occupant ICU rooms (#6, #7, #8, #9, #10, and #11). The bedrails were swabbed on the top and side surface at midpoint where both the patient and staff would be likely to touch (when rising to a seated position for patients and lowering guardrails in the case of staff). There is one keyboard per room that is accessed by the bedside nurse and respiratory therapist assigned to the patient. For each room, the sink is located furthest from the sliding door entrance, kitty corner to the patient bed. The sinks were swabbed along the rim of the sink furthest from the faucet. In addition to handwashing, small amounts of medications may be discarded in the sink during the process of priming intravenous tubing. The occupant (patient) tends not to have any contact with the sink. Samples were stored at − 80 °C until DNA extraction and amplicon sequencing.

### 16S rRNA gene amplicon processing

Swab tips were removed under sterile conditions and subjected to total DNA extraction and concentration via the Qiagen DNeasy Powersoil kit (Qiagen; CA) and Zymo gDNA Clean and Concentrate kit (Zymo; Orange, CA), respectively. In addition, negative extraction controls (unused swab tips) were included to ensure that no DNA contaminated the samples during the extraction and concentration process. From the purified and concentrated DNA the V3-V4 hypervariable region of the 16S rRNA gene was PCR amplified using Kapa Hifi Hotstart Readymix (Kapa Biosystems; Boston, MA) with forward primer 5′-TCG TCG GCA GCG TCA GAT GTG TAT AAG AGA CAG CCT ACG GGN GGC WGC AG-3′ and reverse primer 5′-GTC TCG TGG GCT CGG AGA TGT GTA TAA GAG ACA GGA CTA CHV GGG TAT CTA ATC C-3′ [20] using the following cycling parameters: 95 °C for 3 min, followed by 35 cycles of 95 °C for 30 s, 55 °C for 30 s, 72 °C for 30 s, and a final elongation step of 72 °C for 5 min. Ampure XP beads (Beckman-Coulter; Fullerton, CA) were used to clean resulting amplicons, which were then visualized using a High Sensitivity DNA Kit on a Bioanalyzer (Agilent Technologies; Palo Alto, CA) and quantified via the dsDNA High Sensitivity Kit on a Qubit Fluorometer (Thermo Fisher; USA). Samples were pooled into equal molar proportions and sequenced on the Illumina MiSeq platform (Illumina; San Diego, CA).

### Analysis of the 16S rRNA gene sequences

Sequence reads were processed with the Quantitative Insights Into Microbial Ecology 2 (QIIME2; version 2019.4) [21]. Quality filtering, dereplicating, and chimera filtering were performed using the DADA2 plugin in QIIME2 [22]. Taxonomy classification was generated using the QIIME feature-classifier classify-sklearn feature, with a Naïve Bayes classifier trained on the SILVA database (version 132) [23]. Alpha (Observed Operational Taxonomic Unit (OTU), Shannon index, and Faith’s Phylogenetic Diversity) and beta (Bray Curtis) diversity metrics were produced by QIIME2 core-metrics-phylogenetic pipeline (sampling-depth parameter 15,000) and visualized using the qiime2R (available at https://github.com/jbisanz/qiime2R) and ggplot2 packages in R-Studio (version 1.0.153) [24–26]. Additionally, due to the compositional nature of the data, we opted to test a second beta diversity metric, robust Aitchison PCA, using the DEICODE plugin with taxonomic biplot overlays [27]. Comparison of the relative abundances at the family level was visualized using ggplot2 with the exported QIIME2 taxonomy tables. Differential abundance at genus level (i.e., level 6) was assessed using ANCOM in QIIME2 [28]. The QIIME2 core-feature function, with the maximum fraction set to 90%, was used to define the “core microbiome” for each of the sample sources (bedrail, keyboard, and sink). This identifies the features, a unit of observation (e.g., OTU, amplicon sequence variant), present in at least 90% of samples from each source.

### Statistics

Alpha diversity comparisons among renovation stages and sample sources were assessed by ANOVA with room as a blocking factor. Post hoc Tukey’s honest significant difference (HSD) tests were also conducted to correct for multiple comparisons in R-Studio. Comparison of the relative abundances of the dominant bacterial families and clinically relevant bacterial genera was assessed using Kruskal-Wallis with multiple-hypothesis correction via FDR. Beta-diversity significance was determined using ANOSIM tests with 999 permutations and testing between Bray Curtis dissimilarities was determined with Mann-Whitney *U* tests. Additionally, Spearman correlation coefficients were calculated in R-Studio to identify associations between the days after closure/after opening and the alpha diversity metrics (Observed OTUs, Shannon, and Faith’s PD).

## Results

### Sampling schema and sequencing output

Samples were collected by swabbing hospital ICU bedrails, keyboards, and sinks from six separate rooms (#6, #7, #8, #9, #10, and #11) throughout four stages of the ICU renovation. The first stage was before the ICU closed for renovations, a time when the microbiome would likely be impacted by patients and staff (before closure (BC); 11/16/2016–11/20/16). The second stage was after the ICU closed for renovations (after closure (AC); 11/21/2016–1/10/2017). The third stage began 265 days following the AC stage, a time frame right before the ICU re-opened to patients and staff (before opening (BO); 10/2/2017–10/10/2017). The final stage occurred after the hospital re-opened following the renovations and patients and staff returned (after opening (AO); 10/11/2017–11/25/2017). We chose to examine the microbiota of bedrails and keyboards because they represent high-use patient care surfaces that have previously been shown to capture the interface between healthcare worker and patient microbiota accurately [9, 29]. The sink was selected as a surface because a number of pathogens involved in HAIs are associated with water sources [30, 31].

After DNA extraction, 16S rRNA amplification/sequencing, and quality filtering, 532 samples were included in the analysis (151 from bedrail swabs, 172 from keyboard swabs, and 209 from sink swabs; Table S1). There was a total of 39,557,245 sequence reads with an average number of sequences per sample of 74,355 (± 48,090 S.D.). To account for unequal sequencing depth, data were normalized to a minimum sampling depth of 15,000 sequences per sample. This depth allowed for the majority of samples to be included, while also sufficiently capturing the overall diversity in each sample (Figure S1).

### Alpha diversity at each renovation stage

**Fig. 1:**
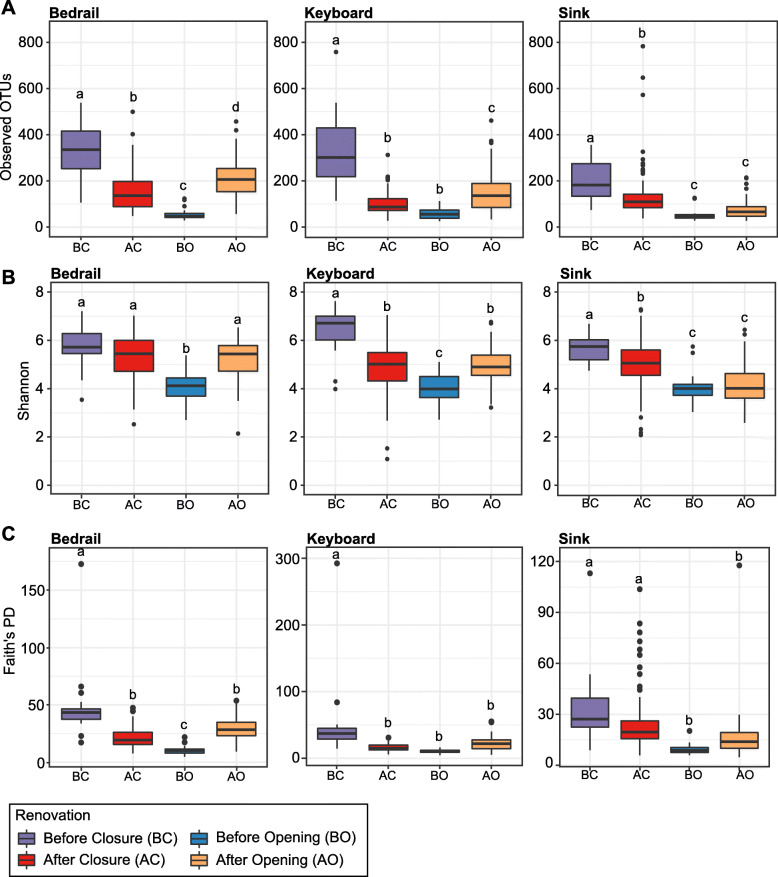
Alpha diversity boxplot showing **a** Observed OTUs, **b** Shannon, and **c** Faith’s PD at each renovation stage for bedrail, keyboard, and sink samples. For each sample source, the alpha diversity indices are shown on the *y*-axis and the renovation stage (before closure, purple; after closure, red; before opening, blue; after opening, orange) are on the *x*-axis. Boxes denote the interquartile range (IQR) between the first and third quartiles, and the horizontal line defines the median. Whiskers represent the smallest (ymin) and largest (ymax) observations within 1.5 times the IQR from the first and third quartiles. Outliers indicated by black circles. Letters shared in common among the renovation stages denote no significant difference (*p* > 0.05) determined by an ANOVA with room as a blocking factor and post hoc Tukey's HSD test

For each sample source (bedrail, keyboard, and sink) and renovation stage (BC, AC, BO, and AO), we examined the alpha diversity of the bacterial communities by calculating the number of Observed OTUs, the Shannon index (a measure that accounts for both richness and evenness), and Faith’s PD (a measure that incorporates phylogenetic differences between species) [25, 26]. Regardless of the metric tested, there were significant (ANOVA; *p* < 0.05) differences in the alpha diversity among the renovation stages (Fig. 1). In all cases, we found that BC had a significantly higher alpha diversity compared to BO (ANOVA; *p* < 0.05), indicating that alpha diversity declined while the ICU was closed. Conversely, for the bedrail and keyboard samples, the Observed OTUs and the Shannon index were significantly higher AO than BO (ANOVA; *p* < 0.05), indicating an increase in diversity after the ICU re-opened. However, the AO stage did not appear to reach the degree of diversity before the ICU closed. Overall, this trend was maintained when observing the alpha diversity metrics from each of the individual ICU room sampled (rooms #6, #7, #8, #9, #10, #11); however, in some cases, not significantly (Figure S2).

Additionally, we compared the alpha diversity of the bedrail, keyboard, and sink samples at each renovation stage (Figure S3). Here, we found significant differences in the alpha diversity among the three sample sources at the majority of renovation stages (ANOVA; *p* < 0.05). However, there were no significant differences in the alpha diversity among the three sample sources at the BO stage. This finding suggests that the decline in diversity during closure is so substantial that differences among the bedrail, keyboard, and sink samples could no longer be detected.

**Fig. 2:**
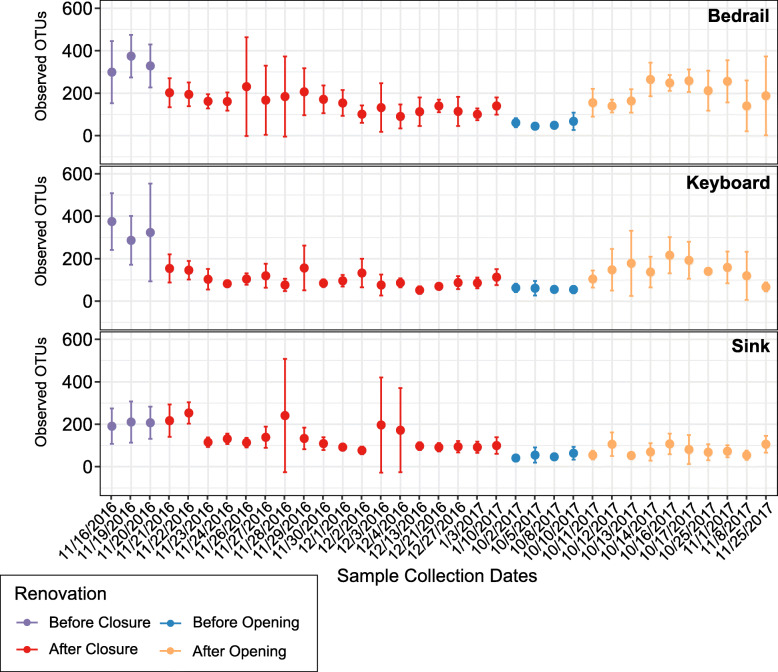
Dotplot of the Observed OTUs (± standard deviation) for bedrail, keyboard, and sink samples at each date throughout the renovation stages. For each sample source, the number of Observed OTUs is shown on *y*-axis and the sampling dates are on the *x*-axis. The samples are colored by renovation stage (before closure, purple; after closure, red; before opening, blue; after opening, orange)

**Fig. 3:**
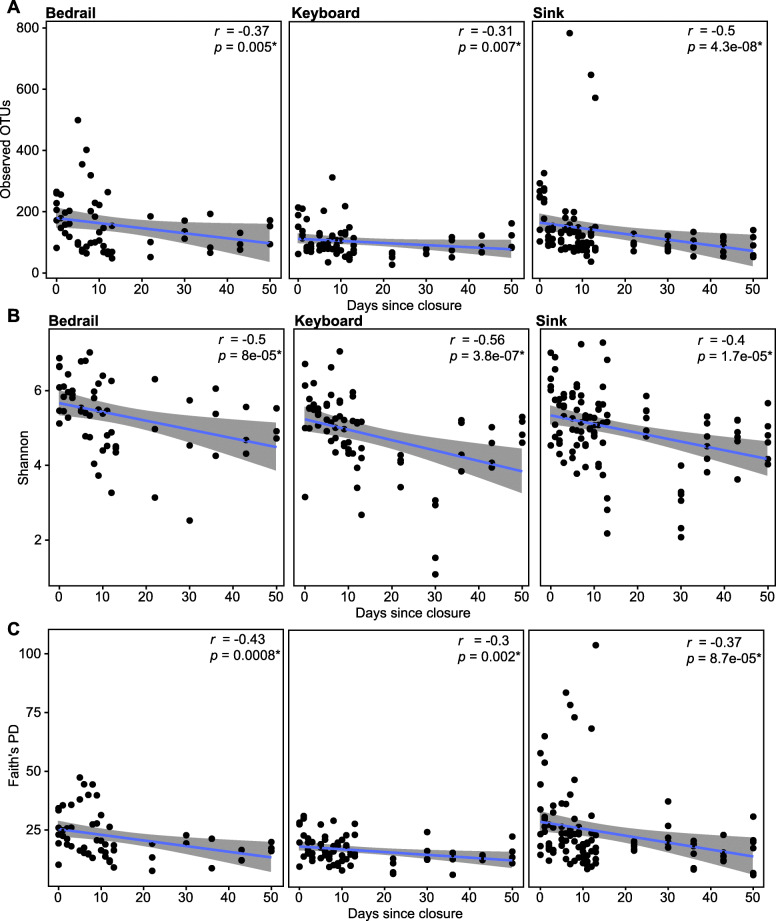
Scatterplot depicting the correlation of **a** Observed OTUs, **b** Shannon, and **c** Faith’s PD with the number of days after hospital ICU closing. For each sample source (bedrail, keyboard, and sink), the alpha diversity indices are shown on the *y*-axis and the days after closing are on the *x*-axis. Blue denotes the linear regression line with the gray shading indicating 95% confidence intervals. Spearman correlation indexes and *p* values are shown in the corner of each panel

To parse the degree to which alpha diversity changed during the ICU renovation further, we examined the three alpha diversity metrics at each sampling date (Fig. 2, S4). For the Observed OTUs, it was apparent that diversity declined after the ICU closed. This trend was also observed for the Shannon and Faith’s PD (Figure S4). In fact, during the AC stage, all of the alpha diversity metrics tested for each source correlated negatively with the number of days following closure (Fig. 3). However, despite observing an increase in alpha diversity after the ICU re-opened, there was not a significant positive correlation with the number of days (Figure S5). These data suggest that maturation of the ICU after opening was dynamic and could not be captured within the 45 days sampled following re-opening.

### Beta diversity by renovation stage

**Fig. 4:**
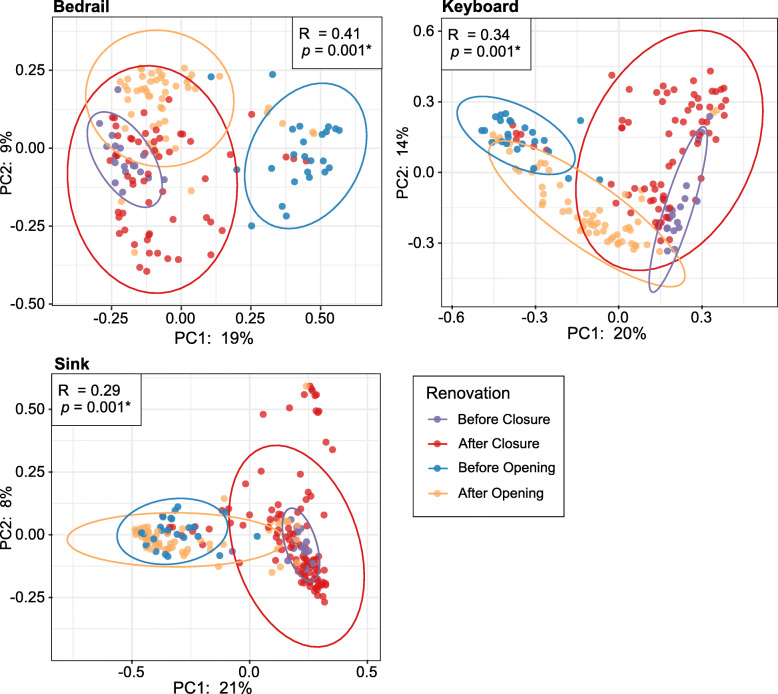
Principal coordinates’ analysis of beta diversity based on Bray Curtis dissimilarities for bedrail, keyboard, and sink samples. Color denotes renovation stages (before closure, purple; after closure, red; before opening, blue; after opening, orange). Ellipses are drawn at 95% confidence intervals for each renovation stage. Significance determined by ANOSIM with 999 permutations for renovation stages and denoted in the corner of each panel *p < 0.05

We next quantified beta diversity using Bray Curtis dissimilarities, a measure widely employed to assess compositional dissimilarity, on each of the sample sources (Fig. 4). We observed a clear and significant differentiation based on renovation stage (Fig. 4; ANOSIM; *p* < 0.05). This was especially evident for the bedrail (ANOSIM; *R* = 0.41, *p* = 0.001) and keyboard (ANOSIM; *R* = 0.34, *p* = 0.001). When utilizing pairwise ANOSIM tests for the bedrail, each renovation stage clustered significantly from one another (ANOSIM; *p* < 0.05), with BC and BO having the highest *R* value (*R* = 0.95). This observation was also true for keyboard samples, with the exception of the AC and BC stages, which were not significantly different (ANOSIM; *R* = 0.09, *p* = 0.06). We also found that the majority of pairwise comparisons for the sink were significant, aside from the BO and AO stages (ANOSIM; *R* = 0.04, *p* = 0.27). Additionally, to further determine if the microbial community was returning to its pre-closure state after re-opening, we compared the Bray Curtis dissimilarities BC with those AO and BO (Figure S6). In each sample source, the BC-AO had a significantly smaller Bray Curtis dissimilarity compared to the BC-BO (Mann-Whitney; *p* < 0.05). This indicates that the ICU microbiome BC was more similar to that of the AO community than BO.

**Fig. 5:**
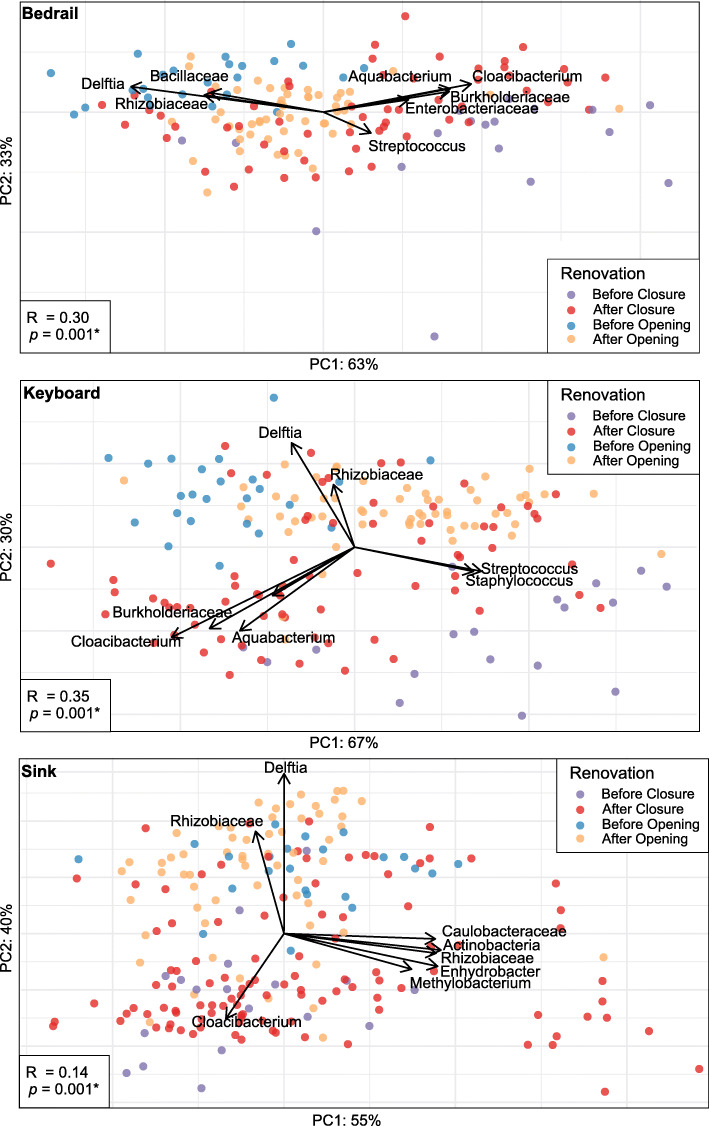
Aitchison compositional biplots for **a** bedrail, **b** keyboard, and **c** sink samples. Color denotes renovation stages (before closure, purple; after closure, red; before opening, blue; after opening, orange). Arrows denote important taxa with regard to sample clusters. Significance determined by ANOSIM with 999 permutations for renovation stages and denoted in the corner of each panel *p < 0.05

Due to the compositional nature of the data, we opted to test a second beta diversity metric, Aitchison PCA. This metric is robust to the high levels of sparsity often found in real microbiome data and can also be used to identify directly the taxa that are likely driving sample clustering [27, 32]. Here, we found significant clustering by renovation stage similar to what was observed by the Bray Curtis metric (Fig. 5 ANOSIM; *p* < 0.05). Moreover, by overlaying biplots corresponding to the taxa that represent the most significant source of variation, we were able to explore the taxonomic factors driving clustering. From these data, we observed that clustering by renovation stage is driven by taxa that have previously been associated with human skin or the environment, especially for the bedrail and keyboard samples [33–36]. Specifically, for the bedrail, we observed that the variation in renovation stages was driven by the dominance of *Delftia*, *Bacillaceae*, and *Rhizobiaceae* in the BO stage and *Streptococcus* in the BC stage. Similarly, for keyboard samples, the variation in renovation stages was driven by the dominance of *Delftia* and *Rhizobiaceae* in the BO stage and *Streptococcus* and *Staphylococcus* in the BC stage.

### Beta diversity of renovation stages by room

We examined the beta diversity via Bray Curtis dissimilarities for each room at the different renovation stages (Figure S7). For each sample source, the rooms were significantly distinct from one another during the BC and AC stages, which indicate a specific microbiome present within each room (ANOSIM; *p* < 0.05). However, at the BO stage, the rooms were no longer significantly different. These data suggest that without the influence of patients and staff, microbial communities across the different ICU rooms become homogeneous.

### Temporal variations in bacterial taxa

**Fig. 6:**
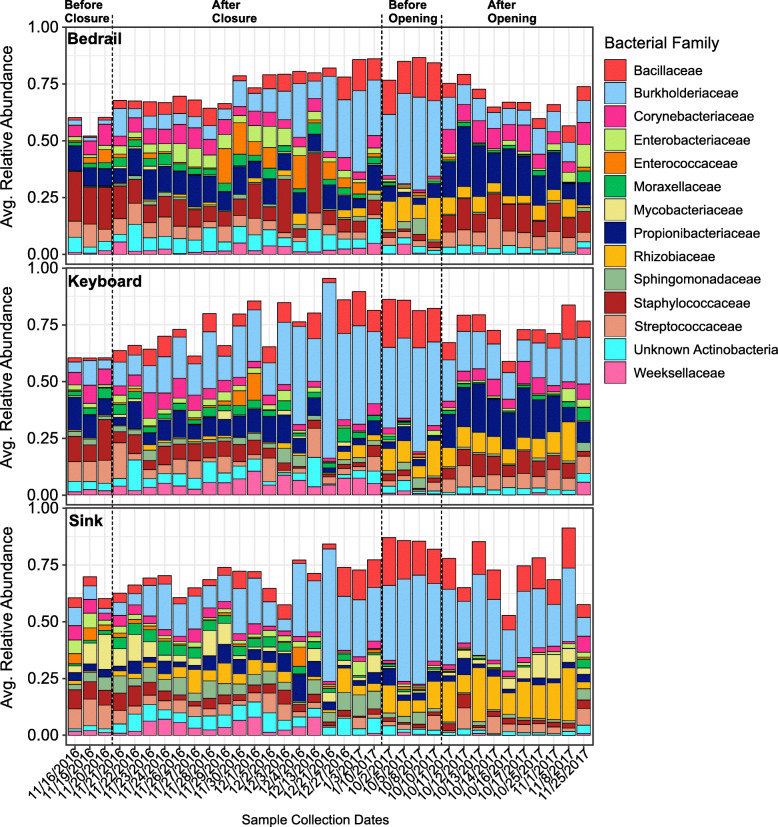
Stacked bar chart of the average relative abundance of the bacterial community composition for samples from bedrails, keyboards, and sinks at each sampling date. For each sample source, the average relative abundance of each of the dominant bacterial families are shown on the *y*-axis and the sampling dates are on the *x*-axis. A dashed line denotes the beginning of the different renovation stages

We next explored the most predominant bacterial families (those that comprised at least 50% of the microbiota in each sample) present within the ICU rooms. When observing the average relative abundances at each date during the renovation, there were transitions that could be visualized among the predominant bacterial families by renovation stage (Fig. 6). Utilizing a Kruskal-Wallis test with multiple-hypothesis correction via FDR, we identified several significant trends. In all sample sources, *Bacillaceae*, *Burkholderiaceae*, and *Rhizobiaceae* were significantly more abundant during the BO stage compared to BC (Kruskal-Wallis; *p* < 0.05; Figure S8). Conversely, for all sample sources in the BC stage, *Corynebacteriaceae*, *Enterobacteriaceae*, *Staphylococcaceae*, and *Streptococcaceae* were significantly more abundant compared to the BO stage. These data are congruent with those presented in the biplot (Fig. 5) in which typical human-associated bacteria were abundant in the established hospital microbiome but were superseded by typical environmental bacteria after ICU closure. Moreover, while the levels of *Staphylococcaceae* increased for the bedrail and keyboard samples in the AO stage compared to BO, they did not return to levels observed in the BC stage. This finding was also true in all sample sources for the abundance of *Bacillaceae* and *Burkholderiaceae*, which decreased in the AO stage, though not to the extent observed BC.

### Clinically significant and core bacterial taxa

To further parse the bacterial taxa present during the various renovation stages, we identified potentially clinically relevant bacteria genera, including *Acinetobacter*, *Bacteroides*, *Burkholderia*, *Clostridium*, *Enterococcus*, *Escherichia-Shigella*, *Klebsiella*, *Mycobacterium*, *Pseudomonas*, *Staphylococcus*, and *Stenotrophomonas* (Figure S9). In all sample sources, *Acinetobacter*, *Bacteroides*, *Escherichia-Shigella*, *Pseudomonas*, and *Staphylococcus* were at significantly higher relative abundance BC than BO (Kruskal-Wallis; *p* < 0.05). Conversely, *Burkholderia* was at a significantly higher relative abundance BO than BC on the bedrail (Kruskal-Wallis; *p* < 0.05). These data are in line with the temporal variations in the relative abundance of the dominant bacterial families described previously (Fig. 6).

We also computed the “core microbiome,” represented by the taxonomic features present in at least 90% of samples from each surface. We found very few taxonomic features that persisted throughout the entire study. However, there were some features that were shared among all three surfaces. On the bedrail, *Bacillaceae*, *Cutibacterium*, *Streptococcus*, *Ralstonia*, *Herbaspirillum*, and *Staphylococcus* were present for the duration of the study. Each of these same taxa, except for *Staphylococcus*, was also identified as core features of the sinks. The keyboard shared four core taxa with the sink and the bedrail throughout the study, including *Bacillaceae*, *Cutibacterium*, *Ralstonia*, and *Herbaspirillum.* There were patterns observed in the relative abundances of the core taxa as we described previously (Figure S10). On the bedrails, BC human-associated bacteria, *Staphylococcus*, *Streptococcus*, and *Cutibacterium* were the dominant core microbiota. However, the abundance of *Staphylococcus* and *Streptococcus* AC declined and were eventually surpassed by *Bacillaceae* in the BO stage. Human-associated bacteria, especially *Cutibacterium*, increased in abundance AO.

### Differentially abundant taxa

**Fig. 7:**
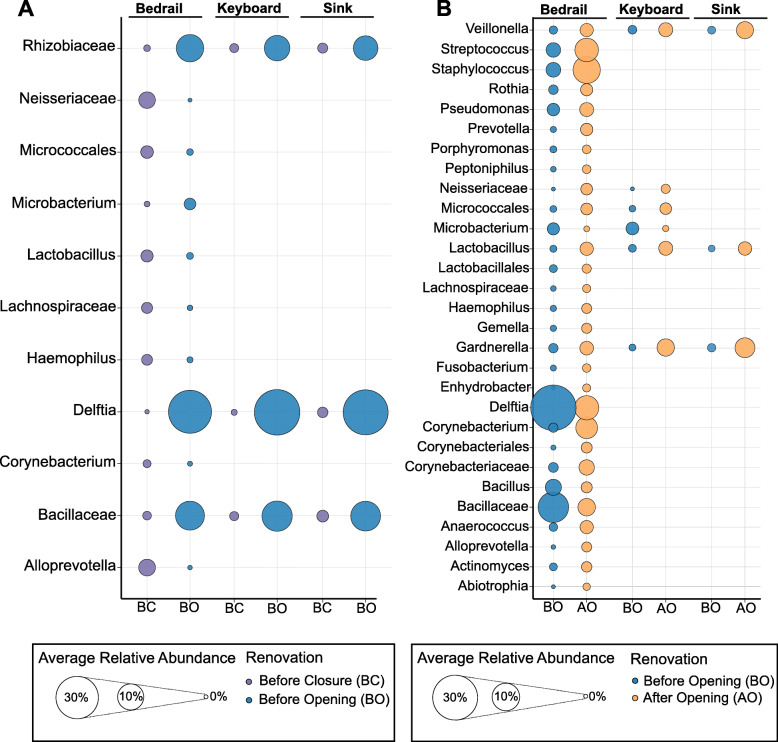
Dotplot of the average relative abundances of the bacterial taxa determined to be differentially abundant between the renovation stages **a** before closure and before opening and **b** before opening and after opening. ANCOM tests were performed to evaluate differential abundance on collapsed feature table at the genus level (i.e., level 6). For each sample source, (bedrail, keyboard, and sink) the taxa determined to be significantly different between stages are shown on the *y*-axis and the renovation stages are on the *x*-axis. The size of the dot reflects the average relative abundance of each taxa and the color denotes the renovation stage (before closure, purple; before opening, blue; after opening, orange)

We next determined the bacterial taxa that significantly differed between relevant renovation stages within each of the sample sources (Fig. 7a). We examined the BC and BO stages, and found 11 taxa that differed significantly. Again, *Bacillaceae*, *Rhizobiaceae*, and *Delftia* (a genera of *Burkholderiaceae*) were more abundant BO in all the sample sources. We also found several human-associated genera, such as *Lactobacillus*, *Haemophilus*, *Corynebacterium*, and *Alloprevotella* that were significantly higher BC in the bedrail samples. Furthermore, we identified 29 taxa that significantly differed between the BO and AO stages (Fig. 7b). However, the majority of these differences were only observed on the bedrail, with only *Veillonella*, *Lactobacillus*, and *Gardnerella* higher in all sample sources AO. This observation suggests that the greatest influence on the bedrail took place AO, and likely resulted from close contact with patients, staff, and visitors.

## Discussion

Understanding how microbial communities colonize and persist on inanimate surfaces in the built environment is critical in evaluating the risks they could pose to public health. These issues are paramount in hospital ICUs wherein surfaces may act as reservoirs for HAIs in a vulnerable patient population [37]. Moreover, as healthcare facilities age and renovations become more commonplace, it is imperative to understand how microbes come to colonize and evolve on surfaces to improve interventions to clean patient care areas. We assessed the evolution of the microbiome of bedrails, keyboards, and sinks in ICU rooms over the course of a renovation. These sites represent common contact points where patients, staff, and visitors interact with the rooms and are likely sites at higher risk of microbe transfers to susceptible patients.

The persistence of important human-pathogens on inanimate surfaces have been reported to range from days to months, depending on a number of factors such as relative humidity, temperature, cleaning procedures, and bacterial genetic composition [38, 39]. In this study, when the ICU closed for renovations and patients and staff were no longer present, the alpha diversity of the bacterial community decreased on all surfaces tested (Fig. 1). Human-associated and potentially clinically significant bacteria (e.g., *Acinetobacter*, *Bacteroides*, *Escherichia-Shigella*, *Pseudomonas*, and *Staphylococcus*) also decreased significantly in their relative abundances after closure (Figs. 1 and 6 and S9). This is in agreement with a previous study that found the influence of an individual’s microbiome on their home surfaces rapidly decays after the individual leaves [40]. As a result, it appears that without regular inoculation, human-associated bacteria do not persist at high relative abundances on ICU surfaces. These bacteria, which are adapted to thrive on the skin of their human host, may be out-competed by resilient environmental-associated species. Some environmental species (e.g., *Bacillus* spp*.*) are capable of persisting in low nutrient environments through actions such as direct antagonism, competition for limited resources, and/or sporulation [41–43]. For instance, cleaning products spiked with non-pathogenic “probiotic” microorganisms (e.g., *B. subtilis*, *B. pumilus*, and *B. megaterium*) were reported to be more effective in the reduction of HAI-associated microorganisms on hospital surfaces when compared to conventional cleaning products [44]. The authors suggest that this is the result of competitive exclusion of the pathogens by the probiotic microorganisms [44]. However, it is important to note that the data presented here are in the form of relative abundances, and thus, do not convey the absolute abundance (e.g., potential infectious dose) of the bacteria present.

We found that during ICU closure, traditional environmental bacterial families, such as *Bacillaceae*, *Burkholderiaceae*, and *Rhizobiaceae* persisted, increasing in abundance days after closure (Fig. 6). Despite decreasing in abundance following hospital re-opening, these bacterial families still remained above the pre-closure level (Fig. 6 and S8). In fact, one bacterial feature, assigned as *Bacillaceae*, was present during the entire study time course on all surfaces tested (Figure S10). Thus, it appears that patients would be in regular contact with these microbes, especially in the weeks after re-opening. While *Bacillaceae*, *Burkholderiaceae*, and *Rhizobiaceae* are generally non-pathogenic and found ubiquitously in water and soil, some have been associated with opportunistic infections. For instance, several members of the *Burkholderiaceae* family (e.g., *Ralstonia pickettii* and *Burkholderia cepacia*) have been linked to outbreaks of HAIs, via contaminated hospital plumbing systems or medications [45–48]. Construction, renovation, and maintenance are known to increase the risk of certain HAIs, particularly those from environmental-associated microorganisms (e.g., *Aspergillus* and *Legionella*) introduced into the hospital setting from dust and soil contamination [49]. However, it is difficult to parse the quantity of environmental-associated bacteria introduced during renovation compared to those that were already present at low or undetectable levels prior to closure. It is also difficult to predict the pathogenic potential of microbes identified from the 16S rRNA sequence data.

When patients and hospital staff returned after re-opening of the ICU, the bacterial communities did not appear to return to the BC status within the time frame of this study. While alpha diversity and the relative abundance of human-associated bacteria (e.g., *Pseudomonas*, *Bacteroides*, and *Staphylococcus*) did increase, they did not achieve the level observed BC (Figs. 1 and 2; S5, S8, S9). Moreover, while the Bray Curtis dissimilarities between BC samples and AO samples were smaller compared to BC and BO samples (Figure S6), the dissimilarity was still quite high and the beta-diversity principle coordinate plots showed very little overlap (Figs. 4 and 5). These findings suggest that, while there was some return to the BC stage after the hospital re-opened, we may not have fully captured the dynamic maturation of the bacterial community within the time frame studied (45 days). Alternatively, it might suggest that the ICU AO is on a different trajectory and that the microbiome may never return to the BC state.

Prior studies show that humans can impact the composition of the bacterial community in the built environment [40]. The influence is largely through transfer of each individual’s specific microbiota onto surrounding surfaces by way of respiration, shedding cells, and direct/indirect skin contact [33, 40, 50, 51]. This phenomenon has been studied in new homes and cohabitation among cadets, where the microbiome of the environment reflects contact with human microbiota in a personalized and unique manner [40, 52]. In a previous study of a hospital microbiome, the authors found that bacteria in patient rooms also resembled the skin microbiota of the patient occupying that room [9]. Similarily, we observed that the hospital ICU rooms were significantly different from their BC state, likely due to the unique microbial communities of the patients admitted to each room and of the personnel working in the area (Figure S7). This is an important consideration as new patients are admitted to these rooms. Prior studies have shown that admission to a room previously occupied by a patient with a HAI-associated microorganism significantly increases the odds of infection by that same microorganism [53–55]. This likely occurs in an early stage of admission before the previous patients’ microbial fingerprint has had time to decay, as we noted the loss of human-associated bacteria and unique signatures AC (Fig. 6, S7).

At the time of ICU re-opening, the ICU occupancy included primarily medical non-surgical, critically ill patients with conditions such as septic shock, cardiac failure, and acute respiratory failure, with an average length of stay between 7 and 10 days. The staff was consistent following re-opening of the ICU, with a nurse to patient ratio of 1-to-1 or 2-to-1. Of the surfaces sampled AO, the increase in human-associated microbes was most evident for the bedrail, with significant increases in genera such as *Veillonella*, *Streptococcus*, *Staphylococcus*, *Rothia*, *Corynebacterium*, and *Gardnerella* (Fig. 7). The bedrail also had the greatest diversity AO, followed by the keyboard and then the sink (Figure S2). This is most likely attributed to the various interactions between the patients/staff and the surfaces. Generally, bedrails have the greatest number of hand-surface interactions by the largest number of different individuals, as they are accessible to staff and visitors. Given their proximity, they are also the surface with the largest magnitude of interaction with the patient. Indeed, previous results found that bedrails consistently resembled the skin microbial community of the current patient [9]. In contrast, just the hospital staff are the primary users of the keyboards and sinks. However, it is not implausible to assume contact transfer from patients to these surfaces byway of the staff [56, 57].

We took advantage of the closure, renovation, and re-opening of an ICU to characterize the microbial diversity and compositional influences on ICU surfaces through different stages of renovation. By profiling different renovation stages, we had a unique opportunity to evaluate temporal changes and determine the influence of the renovation process on the ICU microbiome. We observed clear demarcations at each renovation stage, driven by environmental bacteria AC and human-associated bacteria BC and AO. However, this study is not without limitations. While the 16S rRNA PCR-based methodologies we employed enable us to explore the presence of bacteria that could otherwise be missed by culture alone, there are some inherent challenges. For example, the data produced in this study are in the form of relative abundance and, thus, compositional in nature. Compositional data are constrained to a constant and independent of the microbial load of the original sample, which may be a critical catalyst in patient colonization [32]. The variation in 16S copy number and primer binding and amplification efficiencies can also limit the accuracy in bacterial abundance and diversity estimations [58–62]. Although we employed statistical analyses designed to circumvent some of these challenges (e.g., ANCOM, robust Aitchison PCA) culture-based identification and quantification in tandem with sequence-based methodologies should be explored in future studies. Moreover, this tandem approach may allow for the detection and tracking of specific pathogenic bacteria associated with HAIs, a concept outside the capabilities of the technologies employed here. Nevertheless, we predict that data produced in this study will serve as foundational evidence on the temporal dynamics of the microbes on ICU surfaces and will ultimately help to identify intervention points to reduce the negative impact of the microbiota on patients.

## Conclusions

In this study, we characterized the complex bacterial communities residing on inanimate surfaces in a hospital ICU before, during, and after closure for renovations. These renovations presented us the novel opportunity to capture the transition of the bacterial community from an established microbiome in which patients and staff inhabited the ICU, to one without patients and staff present, and then finally to one where patients and staff returned. We found clear and significant differences in the bacterial community at each stage of renovation. Specifically, alpha diversity was highest before ICU closure and then proceeded to significantly decline during the closure period. After re-opening, the diversity increased, but never reached pre-closure levels. Additionally, we identified significant differences in the microbiota community composition among the renovation stages, which were driven by environmental bacteria after closure and human-associated bacteria after re-opening. Overall, this study provides foundational data on how microbes colonize and evolve on surfaces during times of renovations, a process facing many aging healthcare facilities.

## Supplementary information

**Additional file 1: Figure S1.** Rarefaction curves of (A) Observed OTUs, (B) Shannon, and (C) Faith’s PD for all samples grouped by source and renovation stage. The boxplots showcase the distribution of each alpha diversity metric for each group of samples at each even sampling depth. Boxes denote the interquartile range (IQR) between the first and third quartiles and the horizontal line defines the median. Whiskers represent the smallest (ymin) and largest (ymax) observations within 1.5 times the IQR from the first and third quartiles. **Figure S2:** Alpha diversity bar plot showing (A) Observed OTUs (±standard deviation), (B) Shannon (±standard deviation), and (C) Faith’s PD (±standard deviation) for each room number. Samples are separated by source (bedrail, keyboard, and sink) and colored by the renovation stage (before closure, purple; after closure, red; before opening, blue; after opening, orange). The alpha diversity indices are shown on the y-axis and the room number is on the x-axis. Letters shared in common among the renovations stages for each room denotes no significant difference (*p* > 0.05) determined by an ANOVA with post-hoc Tukey’s HSD test. **Figure S3:** Alpha diversity boxplot showing (A) Observed OTUs, (B) Shannon, (C) Faith’s PD for bedrail, keyboard, and sink samples at each renovation stage. For each renovation stage the alpha diversity indices are shown on the y-axis and sample source (bedrail, pink; keyboard yellow; sink, blue) are on the x-axis. Letters shared in common among the sample sources for each renovation stage denotes no significant difference (*p* > 0.05) determined by an ANOVA with room as a blocking factor and post-hoc Tukey’s HSD test. Boxes denote the interquartile range (IQR) between the first and third quartiles and the horizontal line defines the median. Whiskers represent the smallest (ymin) and largest (ymax) observations within 1.5 times the IQR from the first and third quartiles. Outliers indicated by black circles. Figure S4: Dotplot of the (A) Shannon (±standard deviation) and (B) Faith’s PD (± standard deviation) for bedrail, keyboard, and sink samples at each date throughout the renovation stages. For each sample source the alpha diversity indices are shown on the y-axis and the sampling dates are on the x-axis. The samples are colored by renovation stage (before closure, purple; after closure, red; before opening, blue; after opening, orange). Figure S5: Scatterplot depicting the correlation of (A) Observed OTUs, (B) Shannon, and (C) Faith’s PD with days after hospital ICU re-opening. For each sample source the alpha diversity indices are shown on the y-axis and the days after opening are on the x-axis**.** Blue denotes the linear regression line with the gray shading indicating 95% confidence intervals. Spearman correlation indexes and p-values are shown in either the top right or left hand corner of each panel. Figure S6: Boxplot showing the Bray Curtis dissimilarities between BC-AO and BC-BO for bedrail, keyboard, and sink samples. For each sample source the Bray Curtis dissimilarities are shown on the y-axis and the BC-AO and BC-BO comparisons are on the x-axis. *Significance determined by Mann-Whitney U tests (*p* < 0.05). Boxes denote the interquartile range (IQR) between the first and third quartiles and the horizontal line defines the median. Whiskers represent the smallest (ymin) and largest (ymax) observations within 1.5 times the IQR from the first and third quartiles. Outliers indicated by black circles. Figure S7: Principal coordinates analysis of beta-diversity based on Bray Curtis dissimilarities for (A) bedrail, (B) keyboard, and (C) sink samples for each renovation stage. Color denoted room number. Significance determined by ANOSIM with 999 permutations for rooms and denoted in the corner of each panel *p < 0.05. Figure S8: Bar chart of the relative abundance (±standard deviation) of the bacterial community composition from bedrail, keyboard, and sink samples at each renovation stage. For each sample source the dominant bacterial families are listed on the x-axis and their relative abundance are shown on the y-axis. The bars are colored by the different renovation stages (before closure, purple; after closure, red; before opening, blue; after opening, orange). Letters shared in common between or among renovations stages for each bacterial family denotes no significant difference (*p* > 0.05) determined by Kruskal-Wallis with multiple-hypothesis correction via FDR. Figure S9: Bar chart of the relative abundance (±standard deviation) of clinically relevant bacterial genera from bedrail, keyboard, and sink samples at each renovation stage. For each sample source, the bacterial genera are listed on the x-axis and their relative abundance are shown on the y-axis. The bars are colored by the different renovation stages (before closure, purple; after closure, red; before opening, blue; after opening, orange). Letters shared in common between or among renovations stages for each bacterial genus denotes no significant difference (*p* > 0.05) determined by Kruskal-Wallis with multiple-hypothesis correction via FDR. Figure S10: Line graph of the relative abundance (±standard deviation) of the core bacterial features in bedrail, keyboard, and sink samples at each date throughout the renovation stages. For each sample source the relative abundance of each core feature is listed on the y-axis and the sampling dates are on the x-axis. Only dates marking new renovations stages are shown. Core features are those present in at least 90% of samples from each source.

**Additional file 2: Table S1.** Sample numbers for each source (bedrail, keyboard, and sink) at each renovation stage and for each room.

## Data Availability

All sequences included in this study have been deposited in the NCBI Sequence Read Archive under BioProject accession PRJNA575544.

## Abbreviations

HAIs: Healthcare associated infections
ICU: Intensive care unit
OTU: Operational taxonomic unit
BC: Before closure
AC: After closure
BO: Before opening
AO: After opening
