# Supplementary material for: Temporal variations in bacterial community diversity and composition throughout intensive care unit renovations
Source: Microbiome. 2020 Jun 8;8:86. doi: 10.1186/s40168-020-00852-7 (PMC7278141; doi:10.1186/s40168-020-00852-7)
Supplement: Supplementary file 2 — Additional file 1: Figure S1. Rarefaction curves of (A) Observed OTUs, (B) Shannon, and (C) Faith’s PD for all samples grouped by source and renovation stage. The boxplots showcase the distribution of each alpha diversity metric for each group of samples at each even sampling depth. Boxes denote the interquartile range (IQR) between the first and third quartiles and the horizontal line defines the median. Whiskers represent the smallest (ymin) and largest (ymax) observations within 1.5 times the IQR from the first and third quartiles. Figure S2: Alpha diversity bar plot showing (A) Observed OTUs (±standard deviation), (B) Shannon (±standard deviation), and (C) Faith’s PD (±standard deviation) for each room number. Samples are separated by source (bedrail, keyboard, and sink) and colored by the renovation stage (before closure, purple; after closure, red; before opening, blue; after opening, orange). The alpha diversity indices are shown on the y-axis and the room number is on the x-axis. Letters shared in common among the renovations stages for each room denotes no significant difference (p > 0.05) determined by an ANOVA with post-hoc Tukey’s HSD test. Figure S3: Alpha diversity boxplot showing (A) Observed OTUs, (B) Shannon, (C) Faith’s PD for bedrail, keyboard, and sink samples at each renovation stage. For each renovation stage the alpha diversity indices are shown on the y-axis and sample source (bedrail, pink; keyboard yellow; sink, blue) are on the x-axis. Letters shared in common among the sample sources for each renovation stage denotes no significant difference (p > 0.05) determined by an ANOVA with room as a blocking factor and post-hoc Tukey’s HSD test. Boxes denote the interquartile range (IQR) between the first and third quartiles and the horizontal line defines the median. Whiskers represent the smallest (ymin) and largest (ymax) observations within 1.5 times the IQR from the first and third quartiles. Outliers indicated by black circles. Figure S4: Do [file 40168_2020_852_MOESM1_ESM.pdf]

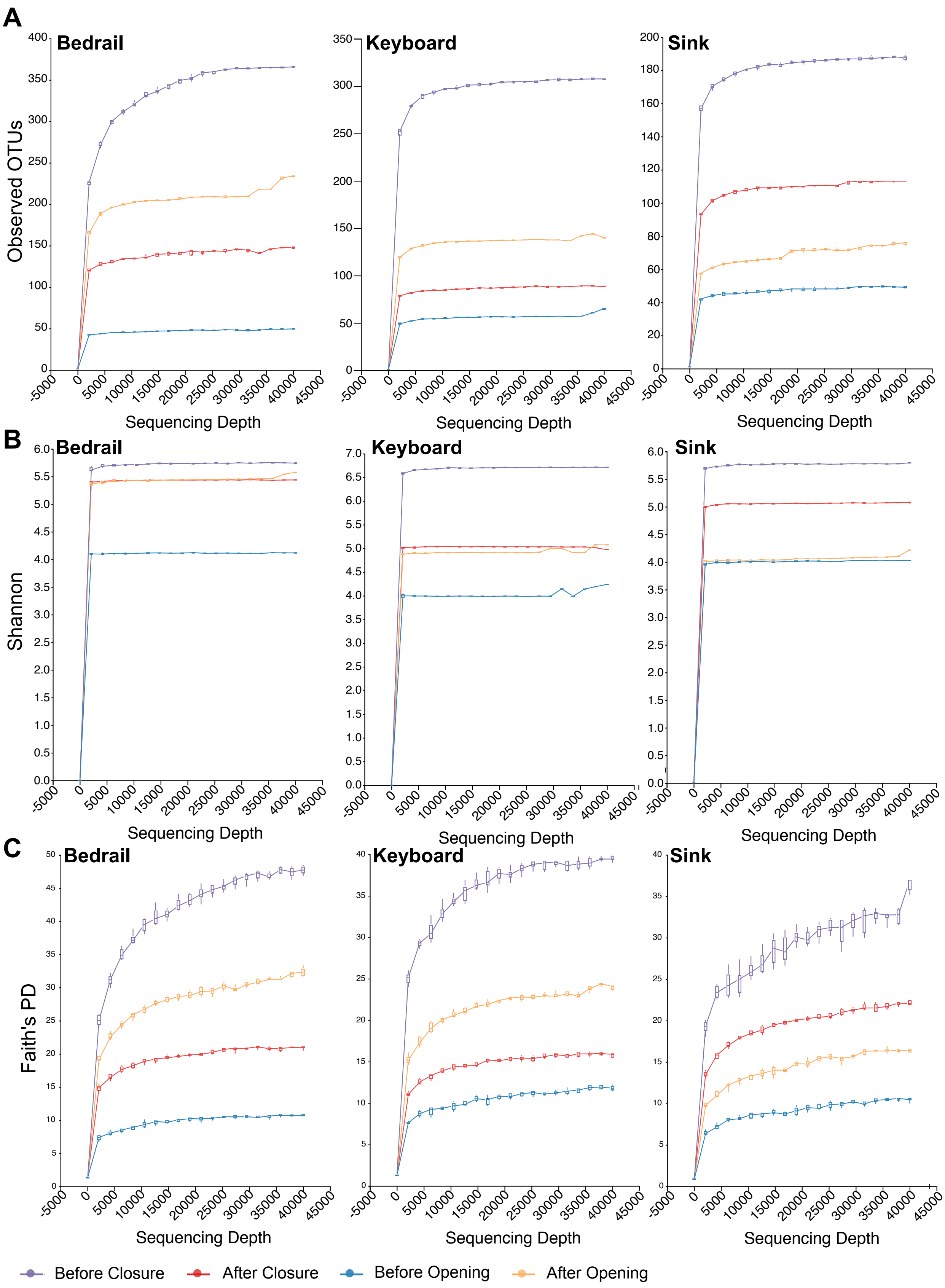
A
Bedrail
Keyboard
Sink
Observed OTUs
Sequencing Depth
B
Shannon
C
Faith's PD
Before Closure
After Closure
Before Opening
After Opening

Figure S1: Rarefaction curves of (A) Observed OTUs, (B) Shannon, and (C) Faith's PD for all samples grouped by source and renovation stage. The boxplots showcase the distribution of each alpha diversity metric for each group of samples at each even sampling depth. Boxes denote the interquartile range (IQR) between the first and third quartiles and the horizontal line defines the median. Whiskers represent the smallest (ymin) and largest (ymax) observations within 1.5 times the IQR from the first and third quartiles.

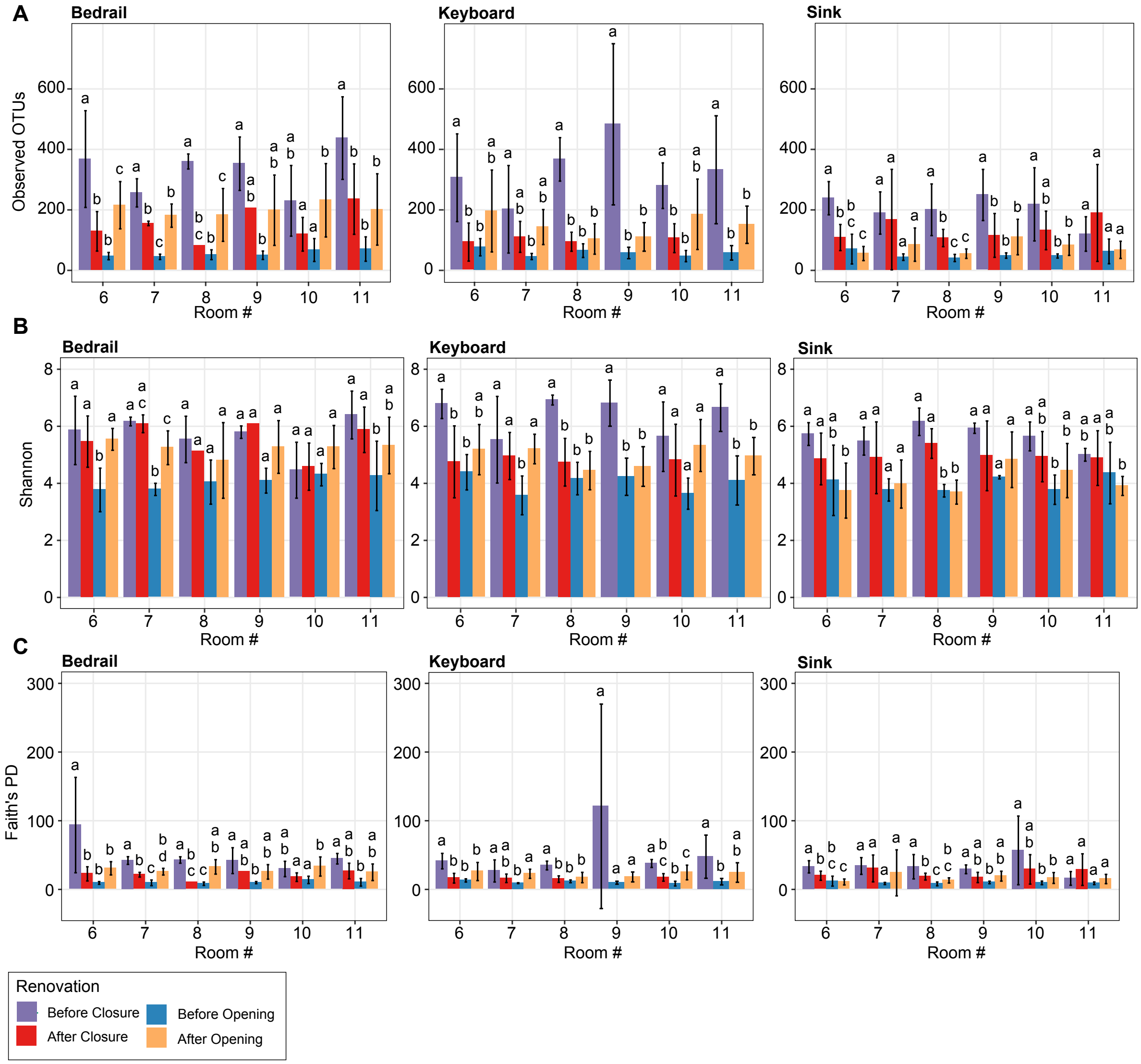

Figure S2: Alpha diversity bar plot showing (A) Observed OTUs (±standard deviation), (B) Shannon (±standard deviation), and (C) Faith's PD (±standard deviation) for each room number. Samples are separated by source (bedrail, keyboard, and sink) and colored by the renovation stage (before closure, purple; after closure, red; before opening, blue; after opening, orange). The alpha diversity indices are shown on the y-axis and the room number is on the x-axis. Letters shared in common among the renovations stages for each room denotes no significant difference (p>0.05) determined by an ANOVA with post-hoc Tukey's HSD test.

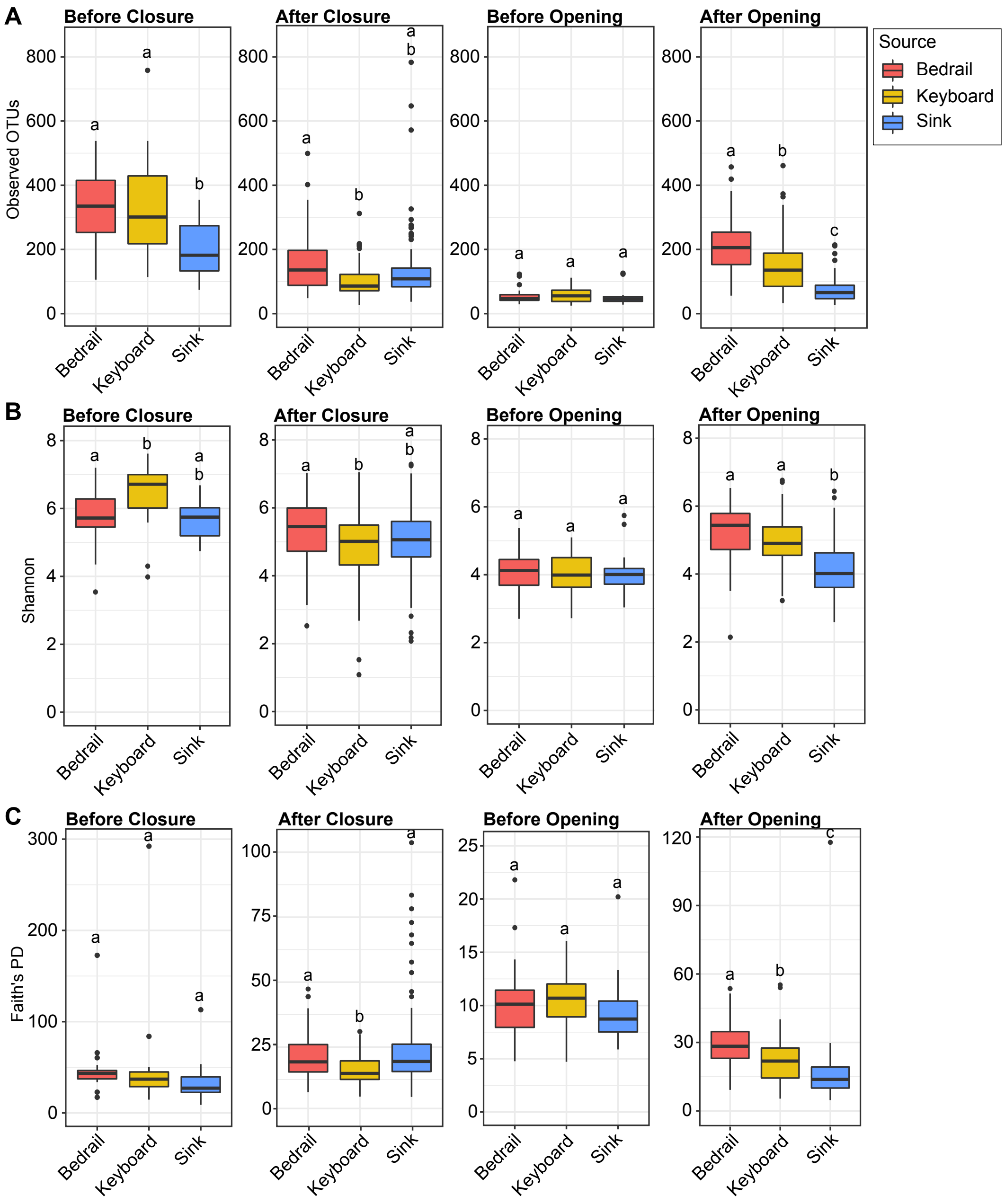

Figure S3: Alpha diversity boxplot showing (A) Observed OTUs, (B) Shannon, (C) Faith's PD for bedrail, keyboard, and sink samples at each renovation stage. For each renovation stage the alpha diversity indices are shown on the y-axis and sample source (bedrail, pink; keyboard yellow; sink, blue) are on the x-axis. Letters shared in common among the sample sources for each renovation stage denotes no significant difference (p>0.05) determined by an ANOVA with

room as a blocking factor and post-hoc Tukey's HSD test. Boxes denote the interquartile range (IQR) between the first and third quartiles and the horizontal line defines the median. Whiskers represent the smallest (ymin) and largest (ymax) observations within 1.5 times the IQR from the first and third quartiles. Outliers indicated by black circles.

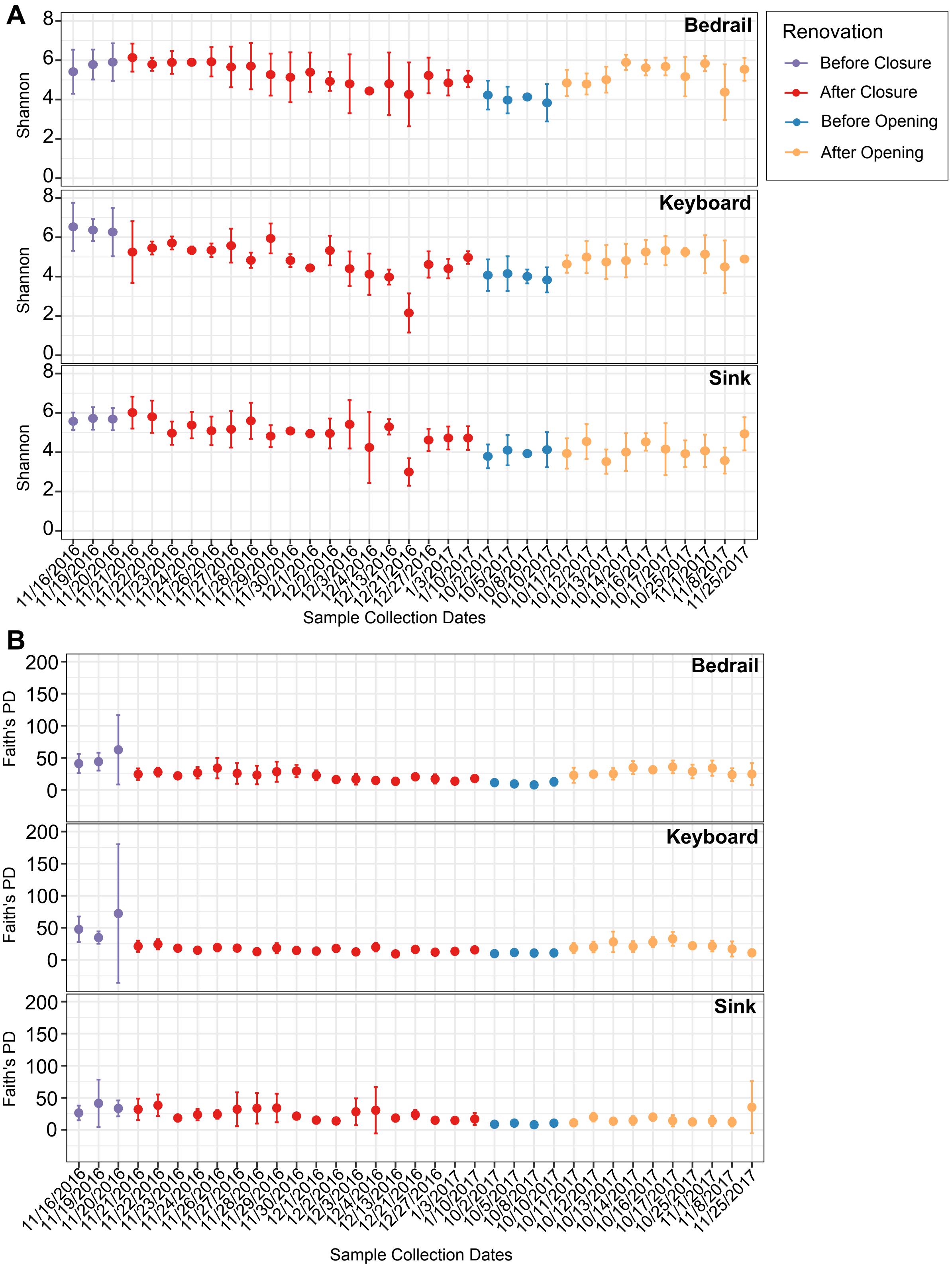

Figure S4: Dotplot of the (A) Shannon (±standard deviation) and (B) Faith's PD (±standard deviation) for bedrail, keyboard, and sink samples at each date throughout the renovation stages.

For each sample source the alpha diversity indices are shown on the y-axis and the sampling dates are on the x-axis. The samples are colored by renovation stage (before closure, purple; after closure, red; before opening, blue; after opening, orange).

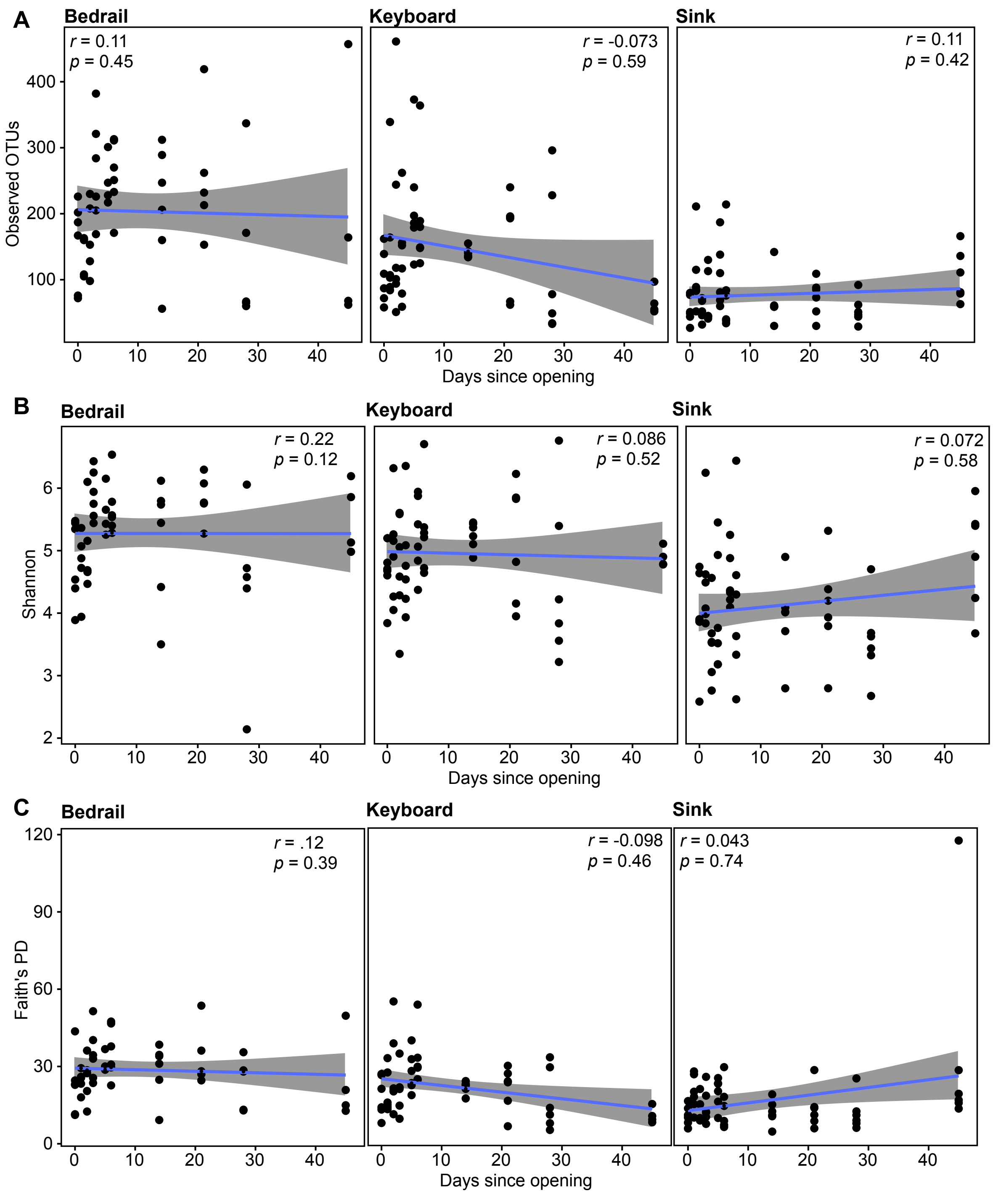

Figure S5: Scatterplot depicting the correlation of (A) Observed OTUs, (B) Shannon, and (C) Faith's PD with days after hospital ICU re-opening. For each sample source the alpha diversity

indices are shown on the y-axis and the days after opening are on the x-axis**.** Blue denotes the linear regression line with the gray shading indicating 95% confidence intervals. Spearman correlation indexes and p-values are shown in either the top right or left hand corner of each panel.

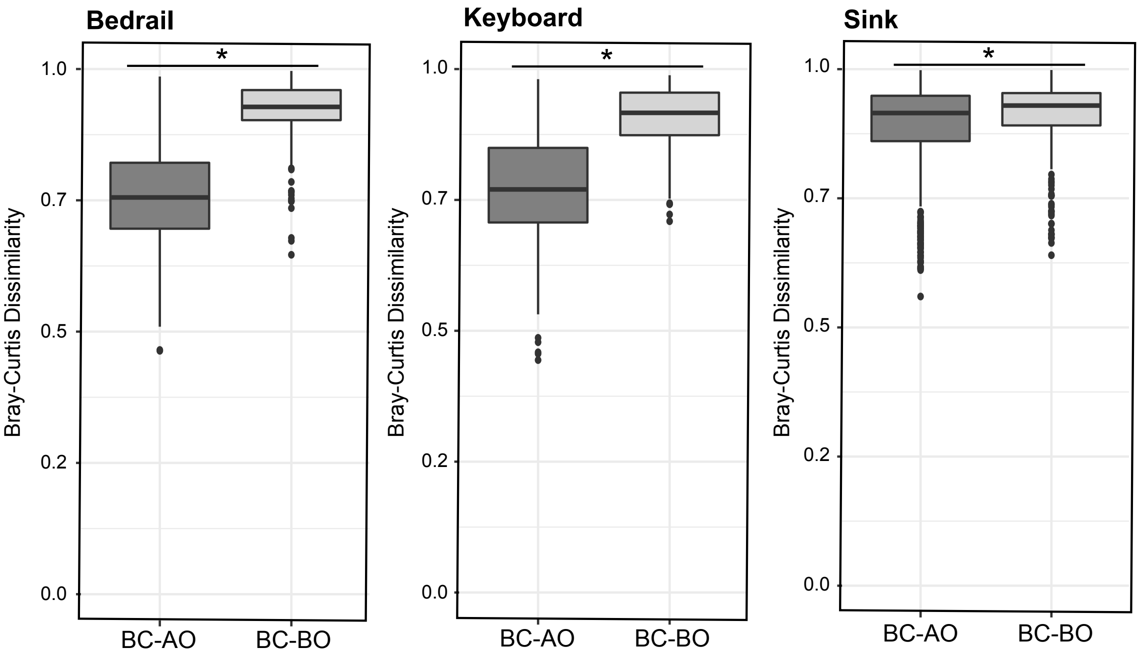

Figure S6: Boxplot showing the Bray Curtis dissimilarities between BC-AO and BC-BO for bedrail, keyboard, and sink samples. For each sample source the Bray Curtis dissimilarities are shown on the y-axis and the BC-AO and BC-BO comparisons are on the x-axis. *Significance determined by Mann-Whitney U tests (p<0.05). Boxes denote the interquartile range (IQR) between the first and third quartiles and the horizontal line defines the median. Whiskers represent the smallest (ymin) and largest (ymax) observations within 1.5 times the IQR from the first and third quartiles. Outliers indicated by black circles.

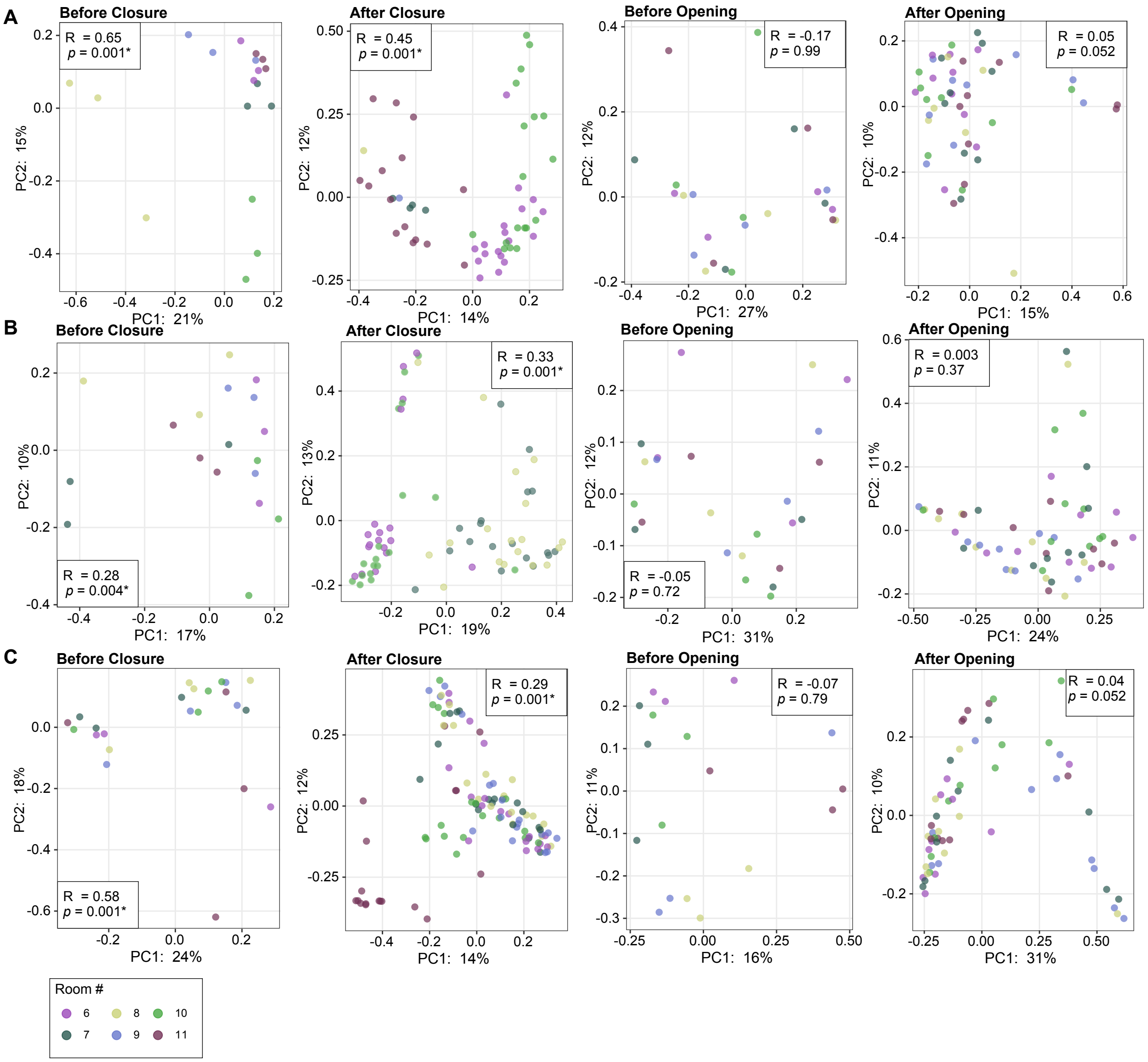

Figure S7: Principal coordinates analysis of beta-diversity based on Bray Curtis dissimilarities for (A) bedrail, (B) keyboard, and (C) sink samples for each renovation stage. Color denoted room number. Significance determined by ANOSIM with 999 permutations for rooms and denoted in the corner of each panel **p*<0.05.

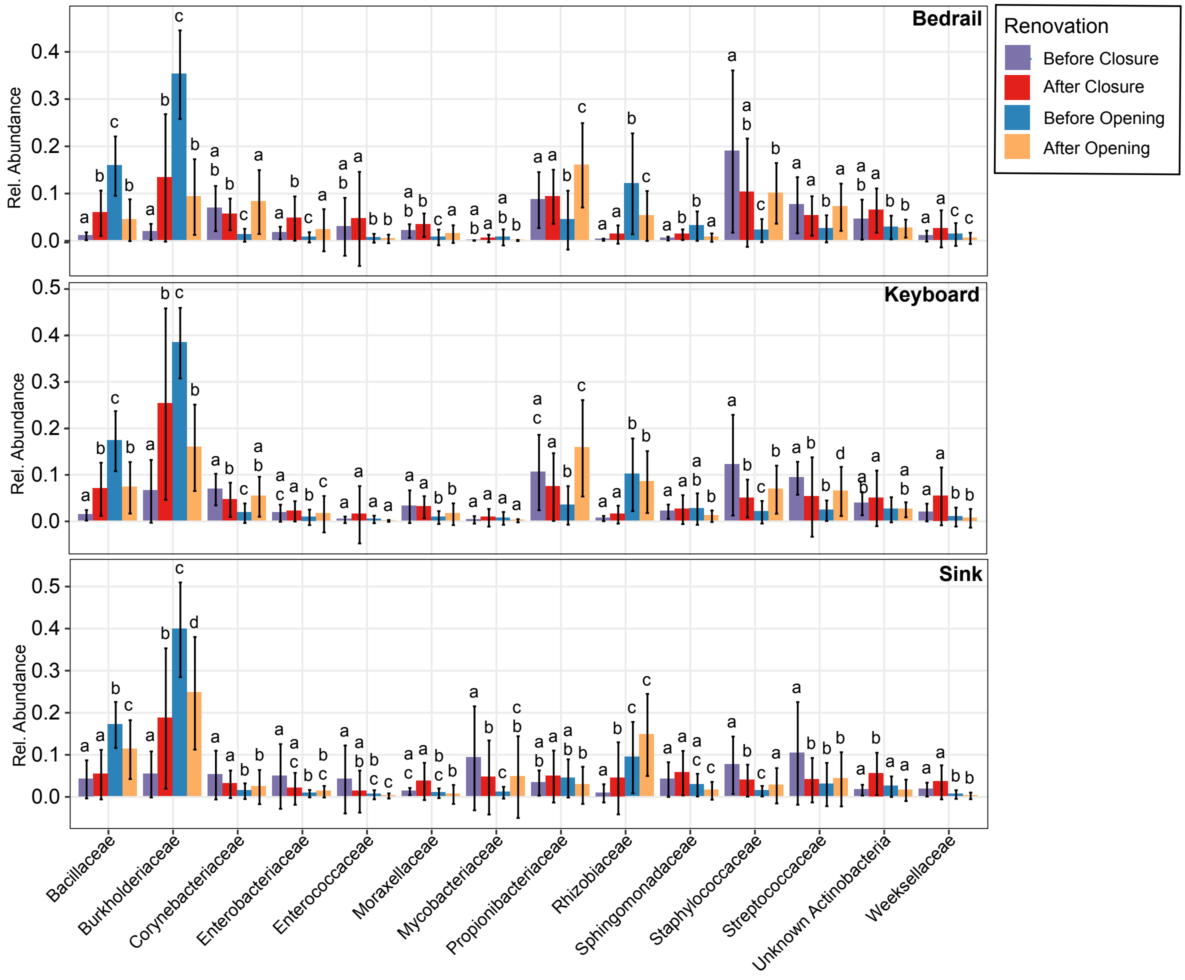

Figure S8: Bar chart of the relative abundance (±standard deviation) of the bacterial community composition from bedrail, keyboard, and sink samples at each renovation stage. For each sample source the dominant bacterial families are listed on the x-axis and their relative abundance are shown on the y-axis. The bars are colored by the different renovation stages (before closure, purple; after closure, red; before opening, blue; after opening, orange). Letters shared in common between or among renovations stages for each bacterial family denotes no significant difference (p>0.05) determined by Kruskal-Wallis with multiple-hypothesis correction via FDR.

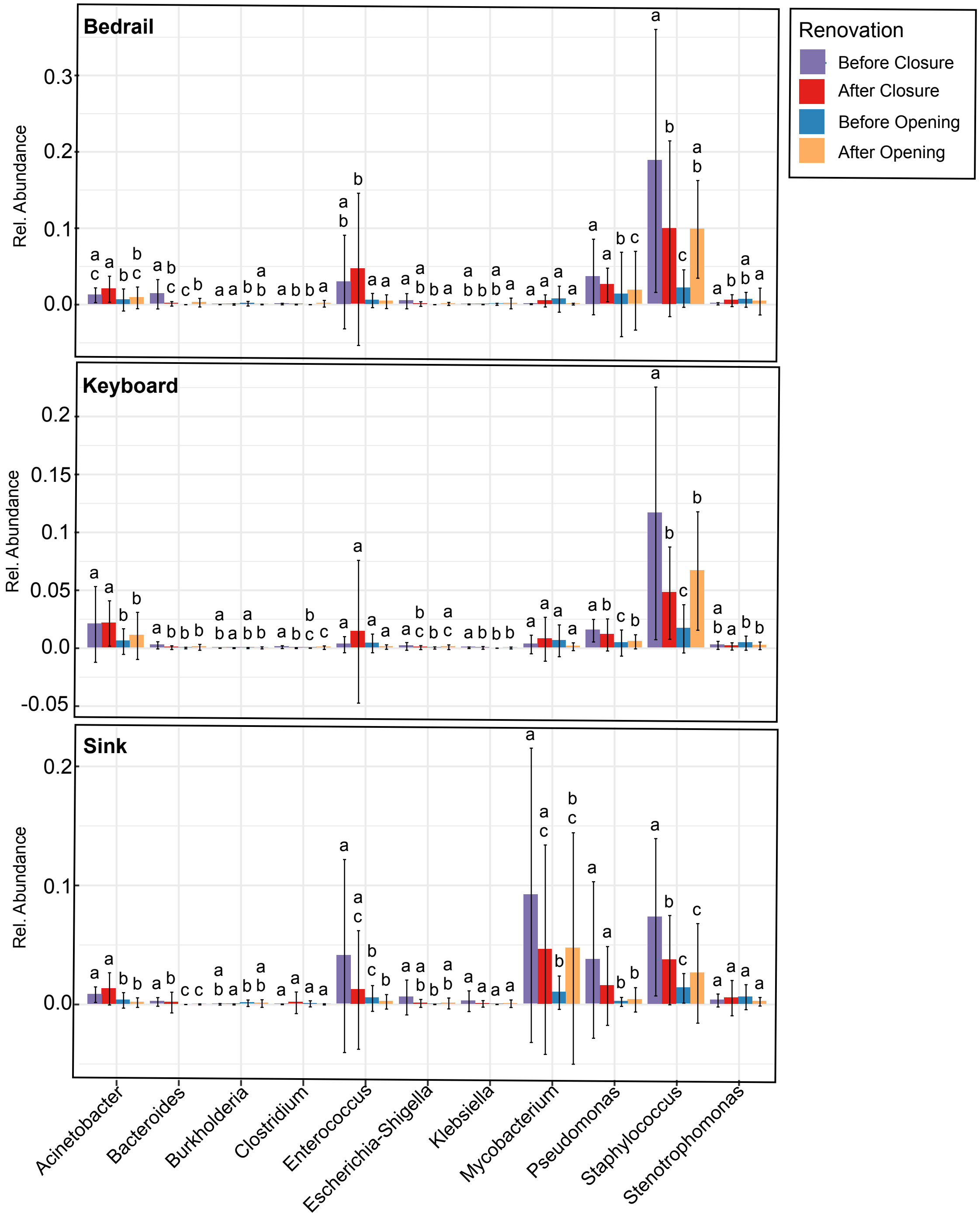

Figure S9: Bar chart of the relative abundance (±standard deviation) of clinically relevant bacterial genera from bedrail, keyboard, and sink samples at each renovation stage. For each sample source the bacterial genera are listed on the x-axis and their relative abundance are shown

on the y-axis. The bars are colored by the different renovation stages (before closure, purple; after closure, red; before opening, blue; after opening, orange). Letters shared in common between or among renovations stages for each bacterial genus denotes no significant difference ($p>0.05$) determined by Kruskal-Wallis with multiple-hypothesis correction via FDR.

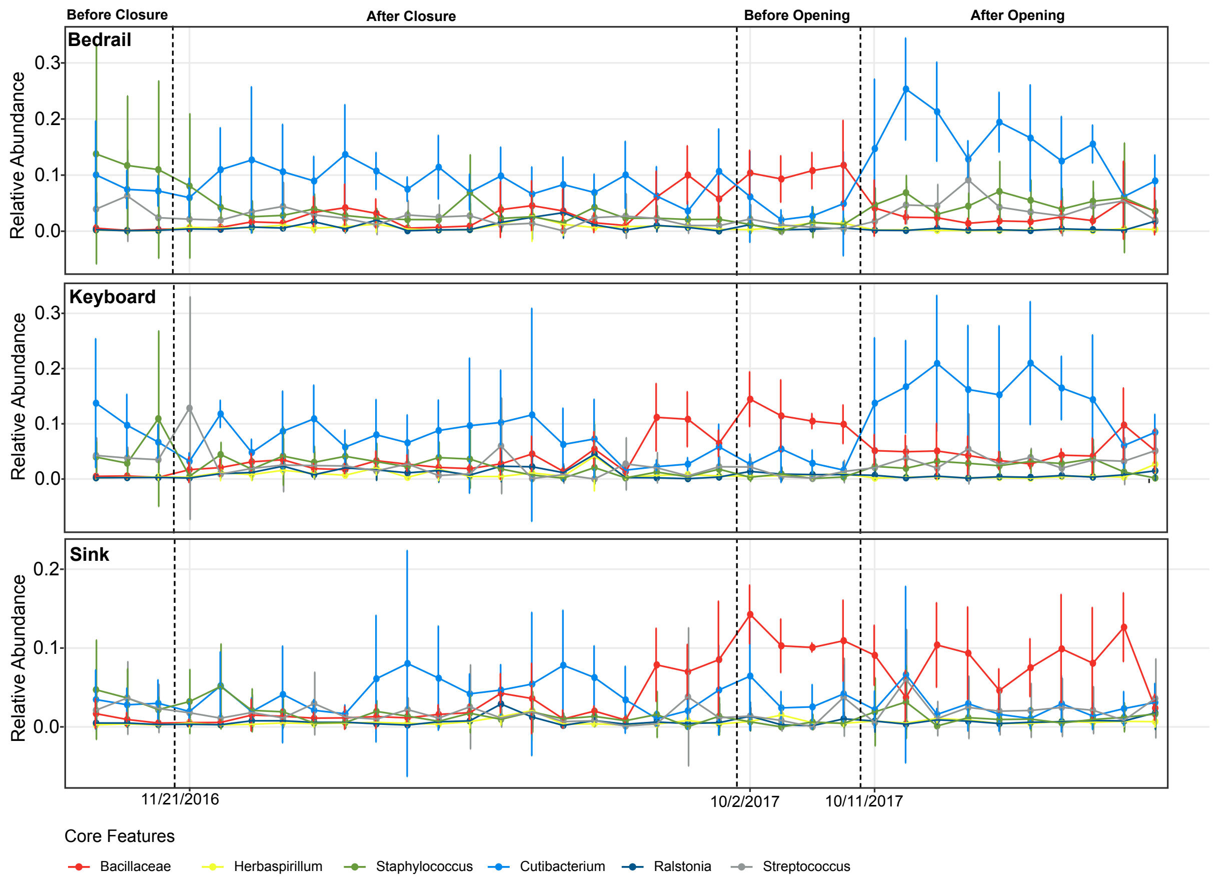

Figure S10: Line graph of the relative abundance (±standard deviation) of the core bacterial features in bedrail, keyboard, and sink samples at each date throughout the renovation stages. For each sample source the relative abundance of each core feature is listed on the y-axis and the sampling dates are on the x-axis. Only dates marking new renovations stages are shown. Core features are those present in at least 90% of samples from each source.
