# Supplementary material for: Temporal variations in bacterial community diversity and composition throughout intensive care unit renovations
Source: Microbiome. 2020 Jun 8;8:86. doi: 10.1186/s40168-020-00852-7 (PMC7278141; doi:10.1186/s40168-020-00852-7)
Supplement: Supplementary file 3 — Additional file 2: Table S1. Sample numbers for each source (bedrail, keyboard, and sink) at each renovation stage and for each room. [file 40168_2020_852_MOESM2_ESM.docx]

**Table S1**: Sample numbers for each source (bedrail, keyboard, and sink) at each renovation stage and for each room.

| **Source** | **Renovation** | **# of samples** | **Room # (n)** |
| --- | --- | --- | --- |
| **Bedrail** | Before Closure | 18 | #6 (3), #7 (3), #8 (3), #9 (3), #10 (3), #11 (3) |
|  | After Closure | 57 | #6 (19), #7 (3), #8 (1), #9 (1), #10 (18), #11 (15) |
|  | Before Opening | 24 | #6 (4), #7 (4), #8 (4), #9 (4), #10 (4), #11 (4) |
|  | After Opening | 52 | #6 (10), #7 (9), #8 (6), #9 (9), #10 (9), #11 (9) |
|  | Total | 151 |  |
| **Keyboard** | Before Closure | 18 | #6 (3), #7 (3), #8 (3), #9 (3), #10 (3), #11 (3) |
|  | After Closure | 72 | #6 (18), #7 (18), #8 (18), #10 (18) |
|  | Before Opening | 24 | #6 (4), #7 (4), #8 (4), #9 (4), #10 (4), #11 (4) |
|  | After Opening | 58 | #6 (10), #7 (10), #8 (10), #9 (9), #10 (10), #11 (9) |
|  | Total | 172 |  |
| **Sink** | Before Closure | 18 | #6 (3), #7 (3), #8 (3), #9 (3), #10 (3), #11 (3) |
|  | After Closure | 108 | #6 (18), #7 (18), #8 (18), #9 (18), #10 (18), #11 (18) |
|  | Before Opening | 23 | #6 (3), #7 (4), #8 (4), #9 (4), #10 (4), #11 (4) |
|  | After Opening | 60 | #6 (10), #7 (10), #8 (9), #9 (11), #10 (10), #11 (10) |
|  | Total | 209 |  |
